# Impact of statins on short and long term mortality in severe community acquired pneumonia in the intensive care unit

**DOI:** 10.1038/s41598-025-25066-5

**Published:** 2025-11-21

**Authors:** Juan Olivella-Gomez, Natalia Sanabria-Herrera, Esteban Garcia-Gallo, Sara Duque, David Luna, Henry Oliveros, Andrew Conway Morris, Alejandro Rodriguez, Djillali Annane, Ignacio Martin-Loeches, Luis Felipe Reyes

**Affiliations:** 1 Clínica Universidad de La Sabana, Chía, Colombia; 2 Unisabana Center for Translational Science, School of Medicine, Universidad de la Sabana, Chía, Colombia; 3 ISARIC, Pandemic Sciences Institute, University of Oxford, Oxford, UK; 4 School of Medicine, Universidad de La Sabana, Chia, Colombia; 5 Division of Perioperative, Acute, Critical Care and Emergency Medicine, Department of Medicine, University of Cambridge, Level 4, Addenbrooke’s Hospital, Hills Road, Cambridge, UK; 6 Division of Immunology, Department of Pathology, University of Cambridge, Cambridge, UK; 7 John V Farman Intensive Care Unit, Addenbrooke’s Hospital, Cambridge, UK; 8 Hospital Joan XXIII de Tarragona, Tarragona, Spain; 9 Department of Intensive Care, Raymond Poincaré Hospital, AP-HP, Garches, France; 10 Simone Veil School of Medicine, Versailles-Saint Quentin University, Paris-Saclay University, Versaillles, France; 11 Institut Hospitalo-Universitaire PROMETHEUS & Fédération Hospitalo- Universitaire SEPSIS, Paris-Saclay University, Saclay, France; 12 INSERM, Garches, France; 13 St James’s University Hospital, Trinity College, Dublin, Ireland; 14 Universidad de La Sabana, Campus Puente del Común, KM 7.5 Autopista Norte de Bogotá, Chía, Colombia

**Keywords:** Mortality, Statin, Pneumonia, Critical care, Propensity score matching, MIMIC, Simulation, Microbiology, Medical research

## Abstract

Severe community-acquired pneumonia (sCAP) carries high mortality. While co-adjuvant therapies remain limited, statins have shown potential benefits. This study simulated a randomised controlled trial (RCT) using real-world data to evaluate the effect of statins on mortality in ICU patients with sCAP. A propensity score matching (PSM) analysis was conducted using the MIMIC-IV database. Kaplan-Meier curves evaluated the impact of statin therapy on 28-, 90-, and 365-day mortality, and Cox regression was used to confirm associations. A total of 4,742 patients met inclusion criteria, including 1,273 who received statins before and/or during the sCAP episode. PSM achieved balanced baseline characteristics between groups. Statin therapy was associated with reduced mortality: average treatment effect − 4.03% (95% CI: -6.9% to -1.0%, *p* = 0.007) using PSM and − 3.7% (95% CI: -6.1% to -1.3%, *p* = 0.002) via inverse probability weighting. Kaplan-Meier analysis showed significantly improved survival for statin users (log-rank *p* < 0.0001). Cox regression confirmed a protective effect (hazard ratio 0.79, 95% CI: 0.72–0.87, *p* = 0.0001). Statin use was associated with reduced acute and long-term mortality in patients with sCAP. These findings highlight the potential role of statins as co-adjuvant therapy, supporting further investigation to optimise treatment protocols.

**Supplementary Information:**

The online version contains supplementary material available at 10.1038/s41598-025-25066-5.

## Introduction

Lower respiratory tract infections (LRTIs) remain a significant global health challenge, especially in their more severe presentations, such as community-acquired pneumonia (CAP)^1,2^. In 2021, it was estimated that there were 344 million episodes of LRTI worldwide, representing 4350 episodes per 100,000 people. LRTI were also responsible for 2,2 million deaths, making them the leading infectious causes of mortality globally^1^. In Europe, pneumonia imposes a substantial economic burden, with annual costs estimated at $10.6 billion^3^. Among hospitalised patients, up to 23% of patients admitted to the hospital develop severe CAP (sCAP), and the mortality rates among these patients are notably higher, with 30-day mortality reaching 27% and rising to 47% within a year^4^.

As the mortality due to sCAP remains extremely high up to a year after the hospital admission, several co-adjuvant treatments have been proposed to improve acute, intermediate, and long-term outcomes. For instance, glucocorticoids and macrolides have been used to reduce the systemic inflammation observed in sCAP patients; however, the results of trials of these treatments have been inconsistent and sometimes contradictory^5,6^. HMG-CoA reductase inhibitors, known as statins, primarily used in clinical practice to control LDL-C levels, have shown benefits such as reducing inflammation, protecting against oxidative stress, slowing cell growth, and modulating the immune response^7^. In a recent adaptive platform trial, simvastatin therapy demonstrated a 95.9% probability of superior to standard care in critically ill patients with COVID-19, improving organ support–free days and mortality compared to those receiving standard care^8^. Furthermore, a meta-analysis revealed that statin use was associated with lower mortality, reduced intensive care unit (ICU) admissions, and a decreased need for mechanical ventilation in the critically ill population, highlighting the potential benefits^7^. However, no trials of statins in sCAP patients have been reported. Therefore, our objective was to simulate a randomised controlled trial (RCT) to evaluate the impact of statins on short- and long-term mortality in patients with sCAP admitted to the ICU.

## Results

A total of 4,742 patients met the inclusion criteria, and 26.8% (1,273/4,742) of the cohort received statin therapy before and/or during and after the sCAP episode. Patients in the statin group were predominantly male (58.4% vs. 53.8%, *p* = 0.005), had a higher median age (72 years vs. 65 years, *p* < 0.001), and a higher Charlson comorbidity index (7 vs. 5, *p* < 0.001). Regarding laboratory variables, patients in the statin group had significantly higher white blood cells minimum (10.2 vs. 9.6, *p* = 0.0001) and maximum (13.2 vs. 12.3, *p* = 0.0002), as well as higher maximum creatinine levels (1.3 vs. 1.1, *p* < 0.001). Furthermore, high-flow nasal cannula therapy (HFNC) was more common in the statin group (4.6% vs. 2.7%, *p* = 0.001). Statin patients showed lower hospital mortality (14.5% vs. 17.6%, *p* = 0.014) and 28-day mortality (18.7% vs. 22.3%, *p* = 0.008). A detailed breakdown of the distribution and p-values for each covariate in both groups is provided in Table 1.

**Table 1:** Baseline characteristics of the cohort.

Characteristic	Statins (*n* = 1273)	Non-statins (*n* = 3182)	Unadjusted *p*-value
Demographic			
Male, n (%)	744 (58.4)	1,714 (53.8)	0.005
Age, median (IQR)	72 (63–80)	65 (53–78)	< 0.001
Charlson comorbidity index, median (IQR)	7 (5–8)	5 (3–7)	< 0.001
***Laboratory variables at admission***,*** median (IQR)***			
Haematocrit max %	33.7 (29.6–38.1)	33.7 (29.7–38.5)	0.613
Haemoglobin max, mg/dL	11 (9.5–12.5)	11 (9.6–12.7)	0.156
Platelets min, cell/mm^3^	201.5 (145–272)	192 (127–273)	0.057
WBC min, cell/mm^3^	10.2 (7.3–13.8)	9.6 (6.4–13.6)	0.0001
WBC max, cell/mm^3^	13.2 (9.4–18)	12.3 (8.4–17.3)	0.0002
Anion gap max, mEq/L	16 (14–19)	16 (13–18)	< 0.001
Bicarbonate min, mEq/L	22 (19–26)	23 (20–26)	0.026
Creatinine max, mEq/L	1.3 (0.9–2.1)	1.1 (0.8–1.7)	< 0.001
BUN max, mg/dL	28 (19–46)	23 (15–39)	< 0.001
Calcium max, mEq/L	8.6 (8.2–9.2)	8.5 (8–9)	< 0.001
Chloride min, mEq/L	100 (96–104)	101 (97–105)	0.0001
Sodium max, mEq/L	139 (137–142)	140 (137–142)	0.24
Potassium max, mEq/L	4.5 (4.1–5.0.1.0)	4.4 (4.0–4.9.0.9)	< 0.001
Glucose min, mg/dL	108 (91–134)	105 (89–124)	0.0001
Lymphocytes max, cell/mm^3^	1.00 (0.63–1.54)	0.98 (0.59–1.56)	0.466
Neutrophils max, cell/mm^3^	10.2 (6.7–14.5)	9.4 (5.9–14.3)	0.006
INR max	1.3 (1.2–1.8)	1.3 (1.1–1.7)	0.004
PT max, sec	14.8 (12.9–19.7)	14.6 (12.9–18)	0.032
PTT max, sec	35 (29.2–50.2)	32.4 (28.2–41)	< 0.001
Urine output, mL	1,425 (870–2255)	1,510 (905–2335)	0.057
***Interventions***,*** n (%)***			
HFCN	59 (4.6)	85 (2.7)	0.001
Invasive ventilation	613 (48.2)	1,452 (45.6)	0.432
**Severity**			
SAPS II, median (IQR)	39 (31–47)	37 (28–46)	< 0.001
ARDS, n (%)	639 (50.2)	1,543 (48.5)	0.304
***Outcomes n (%)***			
28-day mortality	238 (18.7)	710 (22.3)	0.008
90-day mortality	367 (28.8)	1,009 (31.7)	0.06

Abbreviations: ARDS (acute respiratory distress syndrome), BUN (Blood Urea Nitrogen), HFNC (High flux nasal cannula), IQR (Interquartile range), SAPS II (Simplified Acute Physiology Score II), PT (Prothrombin Time), PTT (Partial Thromboplastin Time), WBC (White Blood Cells).

### Propensity score matching

**Fig. 1:**
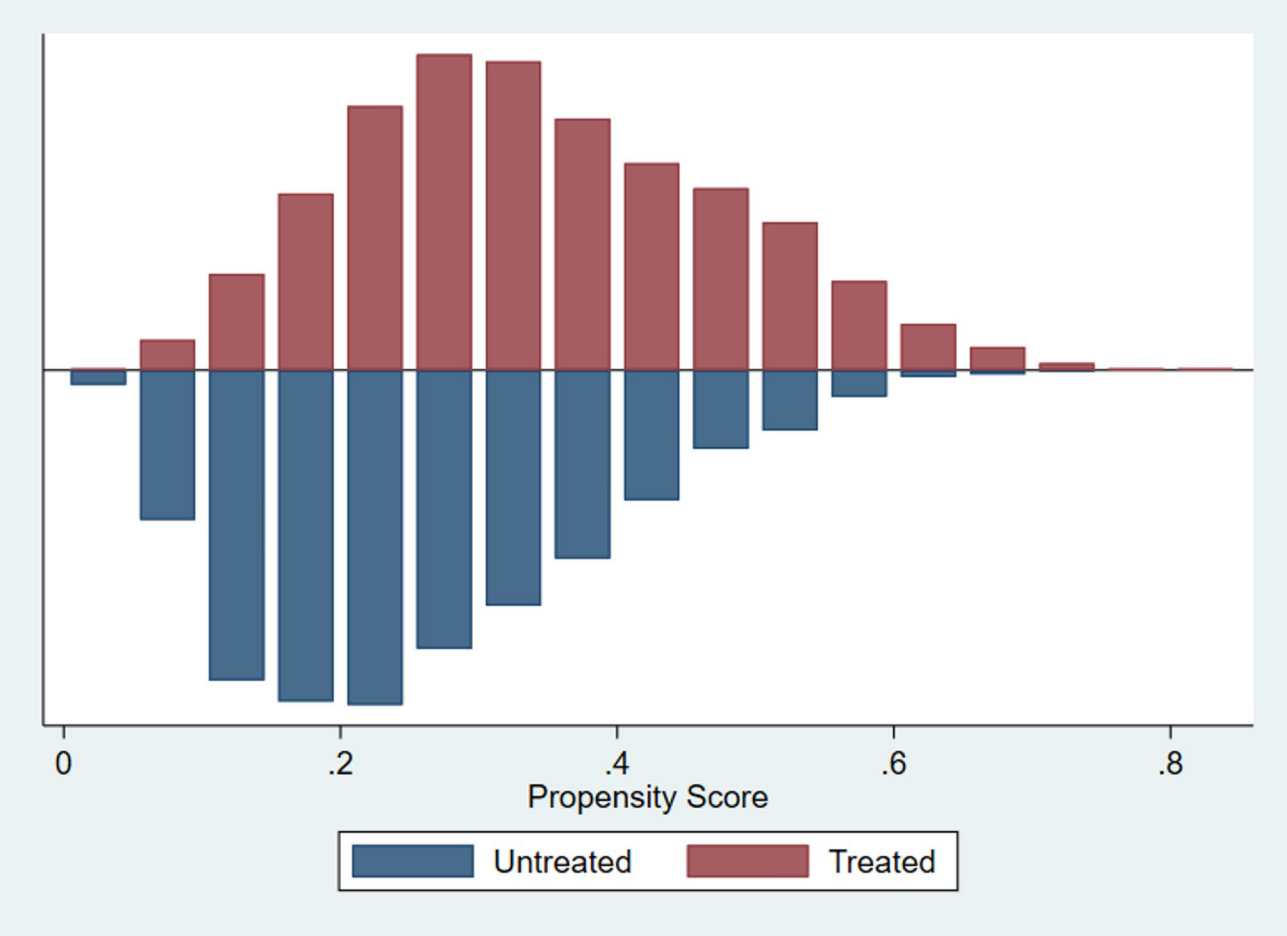
Flowchart of Patient Selection and Total Numbers After Propensity Score Matching. This flowchart illustrates the patient selection process from the MIMIC-IV database. As listed in the panel, 426,489 patients were excluded for various reasons. The final cohort consisted of 4,742 patients, of which 2,783 were included in the matched dataset after performing propensity score matching.

The results in Table 2 summarise the characteristics of patients with sCAP admitted to the ICU, comparing those who received statins with those who did not, both in the unmatched and propensity score-matched cohorts. In the unmatched cohort, patients receiving statins were significantly older (71.01 vs. 64.4 years, *p* < 0.001) and had a higher Charlson Comorbidity Index (6.7 vs. 5.3, *p* < 0.001), indicating a greater burden of comorbidities. The 2:1 ratio was selected as it achieved optimal covariate balance while retaining a substantial number of patients, preserving statistical power without significant loss of participants (Fig. 3). After propensity score matching, baseline characteristics between the statin and non-statin groups were well balanced, as indicated by the reduced standardised mean differences (SMD) in Fig. 1. No significant differences were found in age, Charlson Comorbidity Index, or other relevant variables. In the unmatched cohort, the B statistic was 60.1%, indicating a substantial imbalance between the groups (values above 25% suggest a notable imbalance), and the R statistic was 0.90, reflecting a high degree of variability in the propensity scores. In contrast, after matching, the B statistic was reduced to 8.0%, indicating that the matching process significantly improved the group balance. Additionally, the R statistic increased to 0.96, suggesting a substantial overlap in propensity scores between the matched groups.

**Table 2:** Balance of mean standardised mean differences (SMD) for selected variables before and after propensity score matching.

Characteristic	Unmatched cohort				Matched cohort			
	No statin, (*n* = 3,469)	Statin, (*n* = 1,273)	Standardised differences	*p*-value	No statin, (*n* = 3,059)	Statin, (*n* = 1,203)	Standardised differences	*p*-value
Age	64.40	71.05	0.452	< 0.001	71.30	71.01	−2.0	0.593
Charlson Comorbidity Index	5.31	6.73	0.502	< 0.001	6.68	6.71	0.9	0.826
Generalised Malignancy	0.17	0.14	−0.110	0.002	0.13	0.13	1.6	0.674
Immunosuppression	0.08	0.05	−0.130	< 0.001	0.05	0.05	1.0	0.781
Chronic Kidney Disease	0.17	0.30	0.307	< 0.001	0.29	0.28	2.5	0.591
Transplanted	0.01	0.02	−0.046	0.198	0.01	0.01	−6.5	0.126
Autoimmune Disease	0.03	0.02	−0.027	0.427	0.02	0.02	0.5	0.893
SAPS II	38.22	40.37	0.159	< 0.001	40.4	40.3	−1.0	0.805
SOFA	5.03	5.11	0.022	0.518	5.20	5.11	−2.7	0.499
Creatinine at day 1	1.47	1.78	0.198	< 0.001	1.71	1.77	4.3	0.345
GCS on day 1	13.62	13.71	0.034	0.315	13.71	13.71	0.1	0.974
Temperature	36.9	36.8	−0.063	0.065	36.89	36.87	−4.4	0.282
Viral Pneumonia	0.04	0.04	0.020	0.561	0.05	0.04	−6.7	0.134
ARDS	0.49	0.51	0.036	0.288	0.48	0.50	4.0	0.328

Abbreviations: Simplified Acute Physiology Score II (SAPS II), Sequential Organ Failure Assessment score (SOFA), Glasgow Coma Scale (GCS), Acute Respiratory Distress Syndrome (ARDS).

Average Treatment Effect (ATE) for patients receiving statin treatment compared to those not receiving statins was estimated to be −4.03% (95% CI: −6.9% to −1%), indicating a statistically significant reduction in mortality in those treated with statins (*p* = 0.007). Additionally, the Inverse probability weighting (IPW) method showed a similar result for the ATE estimate of −3.7% (95% CI: −6.1% to −1.3%), significantly reducing mortality (*p* = 0.001). Both methods suggest a beneficial effect of statin therapy on mortality in this cohort Table 3.

**Table 3:** Treatment effect estimates for Mortality.

Method	ATE	Standard Error	z Value	*p* Value	95% CI
Propensity Score Matching	−0.0403	0.0150	−2.67	0.007	[−0.069, −0.010]
Inverse-Probability Weighting	−0.037	0.0121	−3.09	0.002	[−0.061, −0.013]

Abbreviations: Average Treatment Effect (ATE), Confidence Intervale (CI).

### Survival analysis

**Fig. 2:**
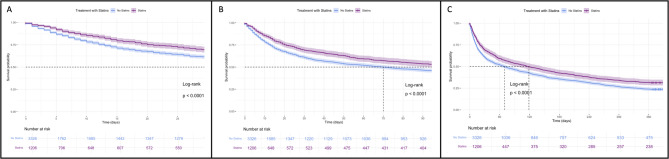
Visualisation of covariate balance using Love plots based on standardised mean differences (SMD) before and after propensity score weighting. The plot shows the standardised % bias across covariates for unmatched (●) and matched (×) groups. The balance improves significantly after matching, as indicated by the reduced bias for the covariates listed.

The Kaplan-Meier analysis shows a significant difference in survival probability between patients treated with statins and those who were not, with a log-rank p-value < 0.0001 (Fig. 2). The survival curve suggests that the statin-treated group has better survival over time than the non-statin group. The Cox regression analysis confirms this difference, with a hazard ratio (HR) of 0.78 (95% CI: 0.71–0.87; *p* < 0.0001) (Table 4),1 indicating that statin-treated patients have a 22% relative risk reduction for mortality compared to those not receiving statins, adjusting for time to death. This result is statistically significant, as demonstrated by the Wald, log-rank, and likelihood ratio tests (all *p* < 0.001).

**Table 4:** Cox regression.

Variable	HR	*p*-value	IC 95% (Lower - Upper)
Statin	0.78	< 0.01	(0.71–0.87)
Age	1.00	0.73	(0.99–1.00)
Charlson Comorbidity Index	1.05	< 0.01	(1.03–1.07)
Chronic Kidney Disease	0.78	< 0.01	(0.69–0.87)
SAPS II	1.02	< 0.01	(1.02–1.03)

Wald Test: 357.4, p < 0.01.

Log-rank Test: 359.1, p < 0.0.

### Sensitivity analyses

The sensitivity analysis evaluated the impact of patient age and macrolide use on mortality using Propensity Score Matching (PSM) and IPW. For patients aged 18–55 years (*n* = 1011), neither PSM (ATE: −0.008; 95% CI: [−0.116, 0.098]; *p* = 0.871) nor IPW (ATE: 0.023; 95% CI: [−0.073, 0.119]; *p* = 0.638) demonstrated a statistically significant effect on mortality. In contrast, for patients aged 56–75 years (*n* = 1959), both PSM and IPW significantly reduced mortality. Among patients aged 56–75 years, the ATEs were − 0.102 (95% CI: [−0.158, −0.045]; *p* < 0.001) via PSM and − 0.093 (95% CI: [−0.139, −0.047]; *p* < 0.001) via IPW. Similarly, for those aged over 75 years, the ATEs were − 0.119 (95% CI: [−0.180, −0.057]; *p* < 0.001) and − 0.107 (95% CI: [−0.161, −0.053]; *p* < 0.001), respectively Supplementary Tables 1–2.

Regarding macrolide use, significant mortality reduction was observed in patients not receiving macrolides (PSM: −0.094; IPW: −0.090; *p* < 0.001), while findings for patients receiving macrolides were inconsistent, with only IPW suggesting a modest benefit (ATE: −0.055; *p* = 0.049). These results highlight the differential effects of age and macrolide therapy on mortality Supplementary Tables 3–4.

## Discussion

This study found that chronic and de novo statin therapy in patients with sCAP significantly reduced 28, 90, and 365-day mortality. This effect demonstrated through PSM and IPW analyses, was further supported by Kaplan-Meier and Cox regression models, which showed sustained mortality reduction over time.

These results align with studies showing how statins prevent severe infections and improve clinical outcomes in bacterial and viral infections through their pleiotropic effects. For instance, Malekinejad et al. described atorvastatin´s anti-inflammatory effects in models of lung inflammation, involving PPARγ receptors reducing alveolar damage and macrophage recruitment, as well as COX-2 expression induced by paraquat^9^. Other in-vivo and in-vitro studies have demonstrated statin´s antioxidant^10^, coagulation-modulating^11^, anti-inflammatory effects^12–14^, and its antibacterial and antifungal properties^15,16^. Erkkilä et al. demonstrated through a murine model how statin treatment reduced mortality from infections caused by pathogens like *Chlamydia* and *Salmonella*, which require cholesterol for intracellular proliferation^16^. Furthermore, Boyd et al. found that simvastatin has effects such as enhanced bacterial clearance and reduced neutrophil infiltration^17^. However, not all findings are consistent, as Radigan et al. concluded in a murine model that rosuvastatin did not improve survival or reduce inflammation in influenza A infections^18^. The mechanisms behind statins’ clinical benefits in pneumonia are not fully understood, but studies suggest a relationship that warrants further exploration.

Clinical evidence also supports statins’ potential to reduce inflammation and infection mortality. In 2001, Liappis et al. conducted the first study on statins in infectious diseases, showing that in-hospital statin use among 388 patients with bacteraemia significantly reduced mortality among statins users (OR 7.6, 95% CI 1.01–57.5)^19^. Subsequently, a 2010 study focused on statin use in pneumonia, including studies from 2005 to 2009 where their use was consistently associated with reduced mortality^20^, aligning with our findings. Furthermore, multiple studies examined statins’ effects on CAP; for instance, a 2019 pilot RCT by Sapey et al. found that simvastatin improved neutrophil function, correlating with better outcomes in CAP patients^21^. Also, a meta-analysis undertaken by Chopra et al. consisting of 13 studies including 254,950 patients revealed that statin use was associated with lower mortality in CAP (OR, 95% CI: 0.62, 0.54–0.71)^22^. Other studies, like the Grudzinska et al. cohort, reported significantly lower in-hospital mortality among CAP patients on statins (OR 0.515; 95% CI 0.403–0.660)^22^. Similarly, Douglas et al. found that statin users had a 33% lower six-month mortality risk following pneumonia with an adjusted HR of 0.67, suggesting that treating 15 CAP patients with statins could prevent one death over six months^23^. However, unlike our study, these studies focused only on short-term benefits and CAP and included only small proportions of patients admitted to the ICU or meeting sCAP criteria; thus, our findings are novel.

The use of statins in viral pneumonia, especially during outbreaks, has also been well-studied. During the 2009 H1N1 epidemic, statin therapy was associated with reduced disease severity among hospitalised patients^24^. Two observational studies further found that statin use was linked to reductions in 30-day mortality (41% and 59%, respectively) in patients hospitalised with influenza infections^25,26^. For COVID-19 specifically, evidence on statins is varied. Rodriguez-Nava et al. reported a reduction in 28-day mortality among COVID-19 who used statins^27^. Additionally, a meta-analysis including seven PSM studies involving 2,398 patients (1,075 on statins) showed that statin use was associated with nearly 40% lower odds of severe illness or death in pneumonia (OR; 95% CI: 0.59; 0.35–0.99)^28^. Finally, another recent RCT found that simvastatin combined with standard therapy in COVID-19 reduced mortality risk^8^. On the other hand, a 2023 meta-analysis of 10 studies with 2,167 COVID-19 patients found no significant differences in mortality (OR 0.96, 95% CI 0.58–1.59, *p* = 0.86) or hospital length of stay (SMD − 0.10, 95% CI −0.78–0.59, *p* = 0.78). However, PCSK9 inhibitors improved mortality outcomes^29^. These results suggest that statins may benefit patients across different types of pneumonia, supporting their potential as an adjunctive treatment to improve survival in severe cases. However, most of these studies did not match the depth of bias control or examine long-term mortality as our study did through PSM analysis, which showed the protective effects of statins persisting up to one year.

In recent years, studies have increasingly used PSM and adjusted models to evaluate the impact of statins on outcomes in pneumonia and COVID-19 patients, addressing potential confounding factors. For instance, a 2020 study by Lohia et al. assessed COVID-19 mortality in 1,014 patients and found that home statin use significantly reduced mortality in the total cohort (OR 0.66; 95% CI 0.46–0.95; *p* = 0.03). This effect was even more pronounced in the PSM cohort of 466 patients, where statin users had a further mortality reduction (OR 0.56; 95% CI 0.37–0.83; *p* = 0.004)^30^. Another study in China by Zhang et al. demonstrated that COVID-19 patients on statins had lower 28-day mortality after PSM, with an adjusted hazard ratio of 0.58. This result was robust across Cox time-varying and marginal structural models^31^. However, none of these studies specifically examined statin use in sCAP, making our study unique as the first to investigate this association in an sCAP cohort. Furthermore, our study includes four times the number of patients compared to previously reported PSM studies and extends the analysis to long-term mortality (up to 365 days), adjusting for a comprehensive range of confounders and competitive mortality to reduce bias. This extensive adjustment and the novel focus on sCAP enhance the robustness and relevance of our findings in a way that previous studies have not achieved.

Our study has strengths and limitations that should be acknowledged. Firstly, applying propensity score matching is a significant methodological advantage, as it helps reduce selection bias and balance confounding variables between statin users and non-users. This approach allows us to better assess the relationship between statin use and mortality outcomes, thereby providing stronger evidence for the observed associations. However, as our analysis is based on retrospective data from the MIMIC-IV database, potential biases inherent to electronic health records, such as missing or inaccurate variables, cannot be handled. Although propensity score matching was applied to mitigate confounding, it is possible that other unmeasured variables or residual confounders were not fully adjusted, which could influence the estimated impact of statin use on mortality. Therefore, our findings should be interpreted as hypothesis-generating or foundation for future research. Additionally, the classification of statin use presents certain challenges. The database lacks details on treatment adherence and dose adjustments during hospitalisation. Notably, observational studies —even when designed to emulate randomized controlled trials—cannot establish definitive causal relationships. These limitations highlight the need for prospective studies and controlled clinical trials to control confounding factors better and assess the effects of continued statin use in patients with sCAP. The consistent results from our analysis lend robustness to our findings, emulating the effects of an RCT by minimising confounding variables and providing a more reliable assessment of statins’ impact on mortality in sCAP patients. Despite the retrospective nature of this study, our approach strengthens the evidence that statins may offer a protective benefit in severe pneumonia cases, warranting further investigation.

In conclusion, this study demonstrates that statin therapy is associated with significantly lower acute and long-term mortality in patients with severe community-acquired pneumonia admitted to the ICU. These findings suggest a potential therapeutic role for statins in improving outcomes in this high-risk population. Our findings underscore the need for further research to understand the mechanisms underlying these effects, especially to determine if benefits persist among patients without dyslipidaemia. As our understanding of statins’ relationship with pneumonia continues to evolve, additional studies are needed to validate these findings and address current knowledge gaps. Ultimately, randomised controlled trials will be essential to confirm statins’ role as co-adjuvants in respiratory infections and inform clinical practice to enhance patient care.

## Methods

This observational cohort study employed the MIMIC-IV database, a comprehensive, high-resolution data archive for critical care research developed by the MIT Lab for Computational Physiology. The database contains deidentified data from approximately 70,000 ICU admissions at Beth Israel Deaconess Medical Center (BIDMC) between 2008 and 2019^32,33^. Research use of MIMIC-IV has been approved by the Institutional Review Boards of BIDMC and MIT, with support from the National Institute of Biomedical Imaging and Bioengineering (NIBIB)^32–34^. The Massachusetts Institute of Technology and Beth Israel Deaconess Medical Center approved the establishment of the MIMIC-IV database, and written informed consent was obtained to collect raw data. Further information about the database can be found elsewhere^32–34^. All personal information was anonymised, and only a random code was used to identify specific patients. Therefore, the Ethics Committee of Clinica Universidad de La Sabana (2023-08-03) and Universidad de La Sabana (Med-519) waived the requirement for informed consent and ethical approval. All methods were carried out in accordance with relevant guidelines and regulations.

### Definitions

#### Severe Community-Acquired pneumonia (sCAP)

Patients with sCAP in the MIMIC-IV database were identified using the ERS/ESICM definition, classifying sCAP as community-acquired pneumonia requiring ICU admission. Immunosuppressed patients, such as those receiving corticosteroids or chemotherapy, undergoing transplantation, or diagnosed with haematological malignancies or HIV with a CD4 count below 200, were excluded^35^.

#### Appropriate antibiotic therapy

Patients who received an antibiotic endorsed by international guidelines **(**IDSA/ATS guidelines). Notably, all patients received at least one β-lactam (including carbapenems) or a fluoroquinolone^36^.

### Study population and data collection

The MIMIC-IV dataset includes detailed information on demographics, clinical observations, laboratory and microbiology results, drug prescriptions, fluid balance, diagnosis codes, procedure codes, and mortality. Additionally, longer-term mortality outcomes were determined by matching MIMIC-IV data with the Social Security Administration’s Death Master File.

**Fig. 3:**
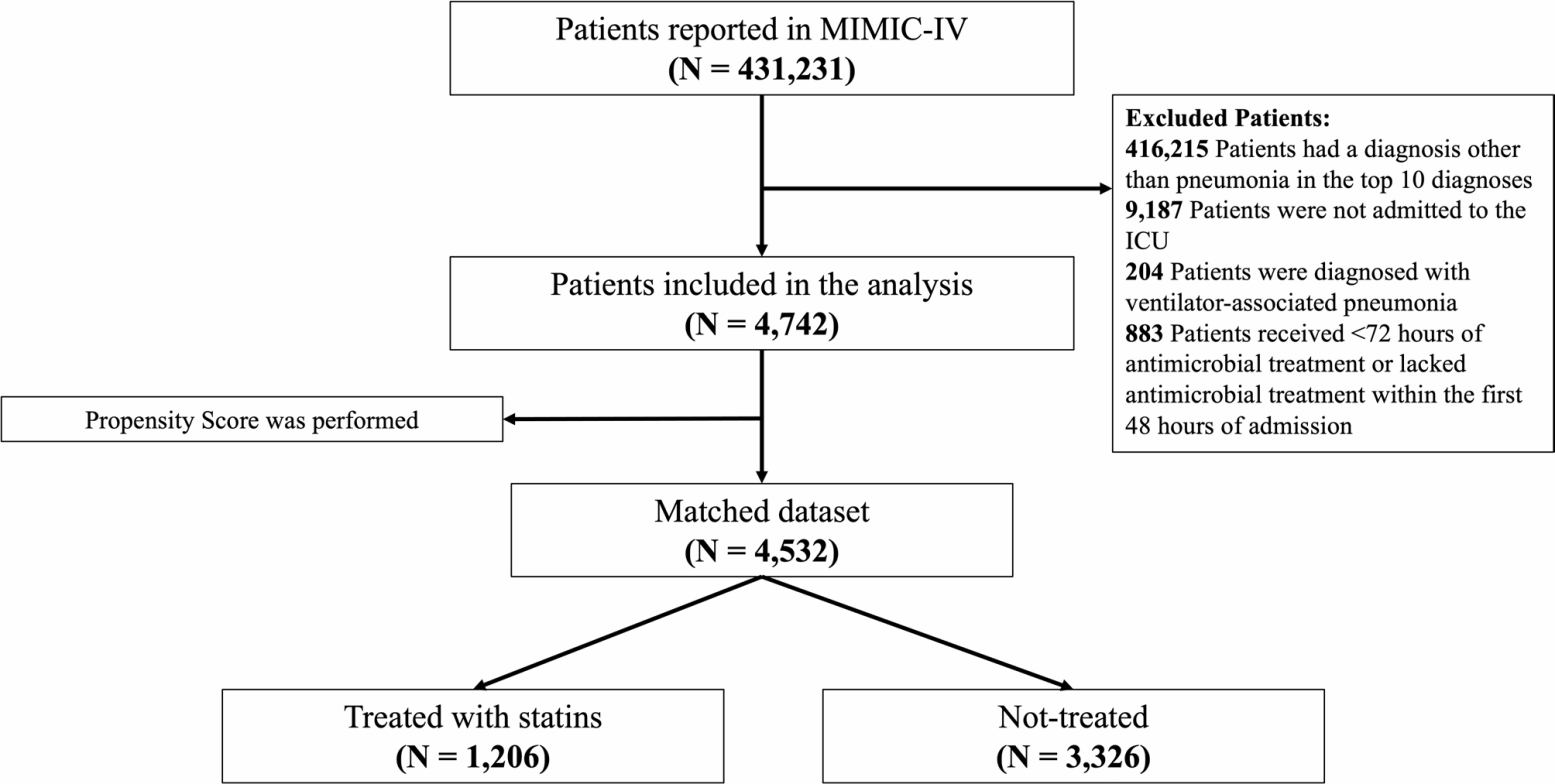
Match graph evaluation. The match graph illustrates the quality of the propensity score matching by depicting the distribution of propensity scores for treated and untreated groups. Evidence of overlap in the propensity scores between groups indicates a well-balanced match.

From the MIMIC-IV database, which contains data on 431,231 patients, those diagnosed with pneumonia were identified using the ten most relevant ICD-9 codes (Supplementary Table 5), a method previously utilised in our research to ensure consistent identification of pneumonia cases^37,38^. Inclusion criteria: initiating appropriate antimicrobial therapy for pneumonia within 48 h of hospital admission and a treatment duration of at least 72 h. Patients with ventilator-associated pneumonia, those not admitted to the ICU, or those with infections other than pneumonia were excluded from the final cohort. Further details on patient selection are provided in Fig. 3.

Then, we created a comprehensive dataset including 255 variables, encompassing demographic information (e.g., gender, age), comorbidities (e.g., myocardial infarction, congestive heart failure), vital signs (e.g., heart rate, blood pressure), laboratory results (e.g., haemoglobin, creatinine levels), and treatments administered (e.g., ventilator support, statins, antibiotic and corticosteroid use).

### Study outcomes

This study’s primary objective was to evaluate statin therapy’s impact on short- and long-term mortality in patients with sCAP admitted to the ICU through simulated RCT. Secondary objectives include identifying the most prevalent comorbidities and describing possible factors influencing mortality.

### Data depuration and variable selection

The dataset underwent several preprocessing steps to ensure data quality and suitability for analysis. These steps included variable selection from the 255 variables extracted from the MIMIC-IV database. To describe the studied population, we selected baseline demographic variables, including age, gender, baseline laboratories not included in severity scores (SOFA and SAPS II), generalised malignancy diagnosis, immunosuppression diagnosis, the Charlson comorbidity index (CCI), chronic kidney disease, transplant status, autoimmune status, creatinine at day-1 and Glasgow coma scale at day-1 of hospitalisation. The CCI was used as a composite measure to capture overall comorbidity burden. Although adjusting for individual comorbidities could provide a more granular approach, not all components of the Charlson system were available. Therefore, we applied the CCI score^39–41^, which has been updated and validated in multiple contemporary cohorts, including critically ill populations. Finally, the treatment with statins was defined as long-term therapy or newly initiated therapy after newly diagnosed dyslipidaemia was initiated before ICU admission but during the concurrent admission. Outcome variables included the number of days to death and whether the patient died during ICU stay or hospitalisation.

### Statistical analysis

Continuous data normally distributed was described using mean and standard deviation (Mean ± SD), with comparisons between groups assessed using the independent samples t-test. Non-normally distributed continuous data were presented as the median and interquartile range (M [Q1, Q3]), and group comparisons were made using the Kolmogorov-Smirnov test. Categorical or nominal data are expressed as the number of cases and the proportion (N [%]), with comparisons conducted using the chi-square test. Rank data are compared using the rank sum test.

Multiple imputation techniques were applied to address missing values in the dataset; variables and patients with more than 30% of missing data were excluded from the analysis. For the remaining cases, binary variables were imputed using the mode, and lineal variables were imputed using the k-means method. PSM was employed to create comparable treatment and control groups by balancing covariates between the two groups. This balance was achieved by weighing each participant through a logistic regression model fitted to estimate propensity scores for each patient. Subsequently, the treatment assignment, statin therapy, was used for the matching method.

**Fig. 4:**
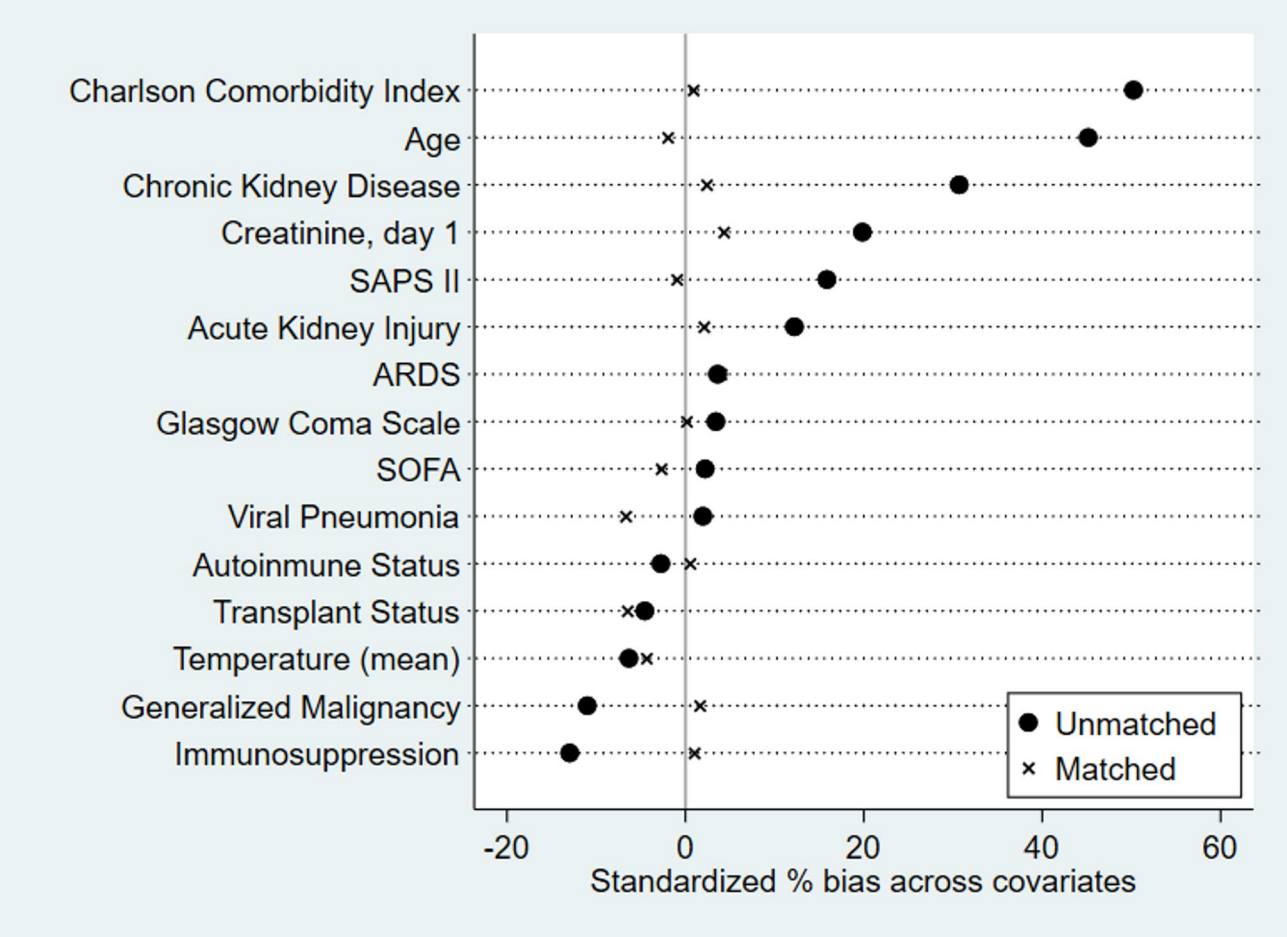
Kaplan-Meier survival curve comparing mortality between patients treated with statins and those not treated. The curve demonstrates the cumulative survival probability over time, with patients in the statin-treated group showing a different survival trajectory compared to the non-treated group. Panel A: Kaplan-Meier survival curve showing patient survival probabilities over 28 days. Panel B: Kaplan-Meier survival curve illustrating survival probabilities over 90 days.

The model was adjusted using the “psmatch2” command in Stata, which facilitates calculating propensity scores and patient matching. A 2:1 matching ratio was applied using the nearest-neighbour method. To ensure adequate group comparability, balance before and after matching was evaluated through SMD and the R and B statistics were calculated^42–44^. SMD scores were calculated to assess covariate adjustment, and different matching ratios (3:1, 2:1, and 1:1) were tested to identify the most efficient approach **(**Fig. 4**)**. Tables based on the matched dataset were presented with bivariate analysis and OR and 95% confidence intervals (CI).

After PSM, the matched dataset was used to fit the survival model. The survival analysis using statin therapy was assessed at 28, 90 and 365 days, with the dataset filtered to include observations at or before these time points. Survival analysis was conducted using Kaplan-Meier curves to evaluate the impact of statin therapy on mortality among patients in the matched dataset. The model assessed the association between statin therapy and mortality while adjusting for potential confounders. Cox proportional hazard regression or other appropriate survival models were utilised for this analysis to calculate the hazard ratio and CI to evaluate the potency of the effect found. Model fit and performance was assessed using standard techniques, including goodness-of-fit tests and the proportional hazards assumption evaluation.

Finally, the ATE was calculated from the matched data. IPW was applied to adjust for confounding by weighting individuals based on their estimated propensity scores for the whole cohort, and sensitivity analyses based on age and macrolide concomitant use. Outcome definitions, including short-term and longer-term mortality, were specified and measured uniformly across both groups. We ensured an adequate follow-up period, with mortality outcomes assessed at 28-, 90- and 365-days post-admission. A p-value of less than 0.05 was considered statistically significant. All analyses were performed using R Studio 2024.04.2 and STATA version 16^45^.

## Supplementary Information

Below is the link to the electronic supplementary material.

Supplementary Material 1

## Data Availability

The datasets analyzed during the current study are derived from the publicly available MIMIC-IV database ([https://doi.org/10.13026/07hj-2a80](https:/doi.org/10.13026/07hj-2a80)), which requires credentialed access and completion of a data use agreement. Additional information generated during the analysis is available from the corresponding author upon reasonable request.

## References

[1] Infections, G. B. D. L. R. & Antimicrobial Resistance, C. Global, regional, and National incidence and mortality burden of non-COVID-19 lower respiratory infections and aetiologies, 1990–2021: a systematic analysis from the global burden of disease study 2021. *Lancet Infect. Dis.* **24**, 974–1002. 10.1016/S1473-3099(24)00176-2 (2024). 38636536 10.1016/S1473-3099(24)00176-2 PMC11339187

[2] Ferreira-Coimbra, J., Sarda, C. & Rello, J. Burden of Community-Acquired pneumonia and unmet clinical needs. *Adv. Ther.* **37**, 1302–1318. 10.1007/s12325-020-01248-7 (2020). 32072494 10.1007/s12325-020-01248-7 PMC7140754

[3] Evans, L. et al. Surviving sepsis campaign: international guidelines for management of sepsis and septic shock 2021. *Intensive Care Med.* **47**, 1181–1247. 10.1007/s00134-021-06506-y (2021). 34599691 10.1007/s00134-021-06506-y PMC8486643

[4] Cavallazzi, R. et al. The burden of Community-Acquired pneumonia requiring admission to ICU in the united States. *Chest* **158**, 1008–1016. 10.1016/j.chest.2020.03.051 (2020). 32298730 10.1016/j.chest.2020.03.051 PMC9458541

[5] Cheema, H. A. et al. Efficacy and safety of corticosteroids for the treatment of community-acquired pneumonia: A systematic review and meta-analysis of randomized controlled trials. *J. Crit. Care*. **80**, 154507. 10.1016/j.jcrc.2023.154507 (2024). 38128217 10.1016/j.jcrc.2023.154507

[6] Giamarellos-Bourboulis, E. J. et al. Clarithromycin for early anti-inflammatory responses in community-acquired pneumonia in Greece (ACCESS): a randomised, double-blind, placebo-controlled trial. *Lancet Respir Med.* **12**, 294–304. 10.1016/S2213-2600(23)00412-5 (2024). 38184008 10.1016/S2213-2600(23)00412-5

[7] Lao, U. S., Law, C. F., Baptista-Hon, D. T. & Tomlinson, B. Systematic review and Meta-Analysis of Statin use and Mortality, intensive care unit admission and requirement for mechanical ventilation in COVID-19 patients. *J. Clin. Med.* **11**10.3390/jcm11185454 (2022). 10.3390/jcm11185454 PMC9501062 36143101

[8] Investigators, R. C. et al. Simvastatin in critically ill patients with Covid-19. *N Engl. J. Med.* **389**, 2341–2354. 10.1056/NEJMoa2309995 (2023). 37888913 10.1056/NEJMoa2309995 PMC10755839

[9] Malekinejad, H., Khoramjouy, M., Hobbenaghi, R. & Amniattalab, A. Atorvastatin attenuates the paraquat-induced pulmonary inflammation via PPARgamma receptors: a new indication for Atorvastatin. *Pestic Biochem. Physiol.* **114**, 79–89. 10.1016/j.pestbp.2014.06.011 (2014). 25175654 10.1016/j.pestbp.2014.06.011

[10] Shishehbor, M. H. et al. Statins promote potent systemic antioxidant effects through specific inflammatory pathways. *Circulation* **108**, 426–431. 10.1161/01.CIR.0000080895.05158.8B (2003). 12860913 10.1161/01.CIR.0000080895.05158.8B

[11] Bickel, C. et al. Influence of HMG-CoA reductase inhibitors on markers of coagulation, systemic inflammation and soluble cell adhesion. *Int. J. Cardiol.* **82**, 25–31. 10.1016/s0167-5273(01)00576-9 (2002). 11786154 10.1016/s0167-5273(01)00576-9

[12] Novack, V. et al. The effects of Statin therapy on inflammatory cytokines in patients with bacterial infections: a randomized double-blind placebo controlled clinical trial. *Intensive Care Med.* **35**, 1255–1260. 10.1007/s00134-009-1429-0 (2009). 19205663 10.1007/s00134-009-1429-0

[13] Fraunberger, P., Grone, E., Grone, H. J. & Walli, A. K. Simvastatin reduces endotoxin-induced nuclear factor kappab activation and mortality in Guinea pigs despite Lowering Circulating low-density lipoprotein cholesterol. *Shock* **32**, 159–163. 10.1097/SHK.0b013e318193c514 (2009). 19008785 10.1097/SHK.0b013e318193c514

[14] Chalmers, J. D., Short, P. M., Mandal, P., Akram, A. R. & Hill, A. T. Statins in community acquired pneumonia: evidence from experimental and clinical studies. *Respir Med.* **104**, 1081–1091. 10.1016/j.rmed.2010.04.005 (2010). 20447815 10.1016/j.rmed.2010.04.005

[15] Catron, D. M. et al. Salmonella enterica serovar typhimurium requires nonsterol precursors of the cholesterol biosynthetic pathway for intracellular proliferation. *Infect. Immun.* **72**, 1036–1042. 10.1128/IAI.72.2.1036-1042.2004 (2004). 14742551 10.1128/IAI.72.2.1036-1042.2004 PMC321618

[16] Erkkila, L. et al. Effect of simvastatin, an established lipid-lowering drug, on pulmonary chlamydia pneumoniae infection in mice. *Antimicrob. Agents Chemother.* **49**, 3959–3962. 10.1128/AAC.49.9.3959-3962.2005 (2005). 16127082 10.1128/AAC.49.9.3959-3962.2005 PMC1195438

[17] Boyd, A. R., Hinojosa, C. A., Rodriguez, P. J. & Orihuela, C. J. Impact of oral Simvastatin therapy on acute lung injury in mice during Pneumococcal pneumonia. *BMC Microbiol.* **12**, 73. 10.1186/1471-2180-12-73 (2012). 22587610 10.1186/1471-2180-12-73 PMC3438118

[18] Radigan, K. A. et al. The effect of Rosuvastatin in a murine model of influenza A infection. *PLoS One*. **7**, e35788. 10.1371/journal.pone.0035788 (2012). 22536437 10.1371/journal.pone.0035788 PMC3335012

[19] Liappis, A. P., Kan, V. L., Rochester, C. G. & Simon, G. L. The effect of Statins on mortality in patients with bacteremia. *Clin. Infect. Dis.* **33**, 1352–1357. 10.1086/323334 (2001). 11565076 10.1086/323334

[20] Brandy Nakashima, M. I. R. & Anzueto, A. Mortensen. The potential role of Statins in pneumonia. *Curr. Respiratory Med. Reviews*. **6**, 155–161. 10.2174/157339810791526193 (2010).

[21] Sapey, E. et al. Simvastatin improves neutrophil function and clinical outcomes in Pneumonia. A pilot randomized controlled clinical trial. *Am. J. Respir Crit. Care Med.* **200**, 1282–1293. 10.1164/rccm.201812-2328OC (2019). 31206313 10.1164/rccm.201812-2328OC PMC6857486

[22] Chopra, V. et al. Is Statin use associated with reduced mortality after pneumonia? A systematic review and meta-analysis. *Am. J. Med.* **125**, 1111–1123. 10.1016/j.amjmed.2012.04.011 (2012). 22835463 10.1016/j.amjmed.2012.04.011

[23] Douglas, I., Evans, S. & Smeeth, L. Effect of Statin treatment on short term mortality after pneumonia episode: cohort study. *BMJ* **342**, d1642. 10.1136/bmj.d1642 (2011). 21471172 10.1136/bmj.d1642 PMC3071610

[24] Fedson, D. S. Treating influenza with Statins and other Immunomodulatory agents. *Antiviral Res.* **99**, 417–435. 10.1016/j.antiviral.2013.06.018 (2013). 23831494 10.1016/j.antiviral.2013.06.018

[25] Vandermeer, M. L. et al. Association between use of Statins and mortality among patients hospitalized with laboratory-confirmed influenza virus infections: a multistate study. *J. Infect. Dis.* **205**, 13–19. 10.1093/infdis/jir695 (2012). 22170954 10.1093/infdis/jir695

[26] Laidler, M. R. et al. Statin treatment and mortality: propensity score-matched analyses of 2007–2008 and 2009–2010 laboratory-confirmed influenza hospitalizations. *Open. Forum Infect. Dis.* **2**, ofv028. 10.1093/ofid/ofv028 (2015). 26034777 10.1093/ofid/ofv028 PMC4438907

[27] Memel, Z. N. et al. Association of Statins and 28-Day mortality rates in patients hospitalized with severe acute respiratory syndrome coronavirus 2 infection. *J. Infect. Dis.* **225**, 19–29. 10.1093/infdis/jiab539 (2022). 34665852 10.1093/infdis/jiab539 PMC8586726

[28] Nowak, M. M., Niemczyk, M., Florczyk, M., Kurzyna, M. & Paczek, L. Effect of Statins on All-Cause mortality in adults: A systematic review and Meta-Analysis of propensity Score-Matched studies. *J. Clin. Med.* **11**10.3390/jcm11195643 (2022). 10.3390/jcm11195643 PMC9572734 36233511

[29] Khalaji, A. et al. Adjunctive therapy with lipid-lowering agents in COVID-19: a systematic review and meta-analysis of randomized controlled trials. *Lipids Health Dis.* **22**, 61. 10.1186/s12944-023-01828-w (2023). 37158917 10.1186/s12944-023-01828-w PMC10165571

[30] Lohia, P., Kapur, S., Benjaram, S. & Mir, T. Association between antecedent Statin use and severe disease outcomes in COVID-19: A retrospective study with propensity score matching. *J. Clin. Lipidol.* **15**, 451–459. 10.1016/j.jacl.2021.03.002 (2021). 33726984 10.1016/j.jacl.2021.03.002 PMC7936125

[31] Zhang, X. J. et al. In-Hospital Use of Statins Is Associated with a Reduced Risk of Mortality among Individuals with COVID-19. *Cell Metab* 32, 176–187 e174, (2020). 10.1016/j.cmet.2020.06.015 10.1016/j.cmet.2020.06.015 PMC7311917 32592657

[32] Johnson, A. et al. (2024). https://physionet.org/content/mimiciv/3.0/.

[33] Johnson, A. E. W. et al. MIMIC-IV, a freely accessible electronic health record dataset. *Sci. Data*. **10**, 1. 10.1038/s41597-022-01899-x (2023). 36596836 10.1038/s41597-022-01899-x PMC9810617

[34] Goldberger, A. L. et al. PhysioBank, PhysioToolkit, and physionet: components of a new research resource for complex physiologic signals. *Circulation* **101**, E215–220. 10.1161/01.cir.101.23.e215 (2000). 10851218 10.1161/01.cir.101.23.e215

[35] Martin-Loeches, I. et al. ERS/ESICM/ESCMID/ALAT guidelines for the management of severe community-acquired pneumonia. *Intensive Care Med.* **49**, 615–632. 10.1007/s00134-023-07033-8 (2023). 37012484 10.1007/s00134-023-07033-8 PMC10069946

[36] Metlay, J. P. et al. Diagnosis and treatment of adults with Community-acquired Pneumonia. An official clinical practice guideline of the American thoracic society and infectious diseases society of America. *Am. J. Respir Crit. Care Med.* **200**, e45–e67. 10.1164/rccm.201908-1581ST (2019). 31573350 10.1164/rccm.201908-1581ST PMC6812437

[37] Reyes, L. F. et al. Impact of macrolide treatment on long-term mortality in patients admitted to the ICU due to CAP: a targeted maximum likelihood Estimation and survival analysis. *Crit. Care*. **27**, 212. 10.1186/s13054-023-04466-x (2023). 37259125 10.1186/s13054-023-04466-x PMC10230128

[38] Serrano-Mayorga, C. C. et al. A targeted likelihood Estimation comparing cefepime and piperacillin/tazobactam in critically ill patients with community-acquired pneumonia (CAP). *Sci. Rep.* **14**, 13392. 10.1038/s41598-024-64444-3 (2024). 38862579 10.1038/s41598-024-64444-3 PMC11166966

[39] Charlson, M. E., Pompei, P., Ales, K. L. & MacKenzie, C. R. A new method of classifying prognostic comorbidity in longitudinal studies: development and validation. *J. Chronic Dis.* **40**, 373–383. 10.1016/0021-9681(87)90171-8 (1987). 3558716 10.1016/0021-9681(87)90171-8

[40] Quan, H. et al. Updating and validating the Charlson comorbidity index and score for risk adjustment in hospital discharge abstracts using data from 6 countries. *Am. J. Epidemiol.* **173**, 676–682. 10.1093/aje/kwq433 (2011). 21330339 10.1093/aje/kwq433

[41] Radovanovic, D. et al. Validity of Charlson comorbidity index in patients hospitalised with acute coronary syndrome. Insights from the nationwide AMIS plus registry 2002–2012. *Heart* **100**, 288–294. 10.1136/heartjnl-2013-304588 (2014). 24186563 10.1136/heartjnl-2013-304588

[42] Austin, P. C. Balance diagnostics for comparing the distribution of baseline covariates between treatment groups in propensity-score matched samples. *Stat. Med.* **28**, 3083–3107. 10.1002/sim.3697 (2009). 19757444 10.1002/sim.3697 PMC3472075

[43] Rubin, D. Using propensity scores to help design observational studies: application to the tobacco litigation. *Springer Nat. Link.* **2**, 169–188. 10.1023/A:1020363010465 (2001).

[44] Paul, R. & Rosenbaum, D. B. R. Constructing a control group using multivariate matched sampling methods that incorporate the propensity score. *Am. Stat.* **39**, 33–38. 10.2307/2683903 (1985).

[45] RStudio *Integrated Development for R v. 2024.04.2 (RStudio* (PBC, 2024).

