# Supplementary material for: Impact of statins on short and long term mortality in severe community acquired pneumonia in the intensive care unit
Source: Sci Rep. 2025 Nov 21;15:41212. doi: 10.1038/s41598-025-25066-5 (PMC12638856; doi:10.1038/s41598-025-25066-5)
Supplement: Supplementary file 1 — Supplementary Material 1 [file 41598_2025_25066_MOESM1_ESM.docx]

**Supplementary Figure 1**. Comparison of standardised mean difference (SMD) adjustment across different ratio matching strategies. Panels A, B, and C: SMD adjustment for 3:1, 2:1, and 1:1 matching strategy at 28 days, respectively. Panels D, E, and F: SMD adjustment for 3:1, 2:1, and 1:1 matching strategy at 90 days, respectively. This analysis was performed as part of the methodology to select the matching strategy that provided the best covariate balance, ensuring optimal adjustment and comparability between treatment groups.


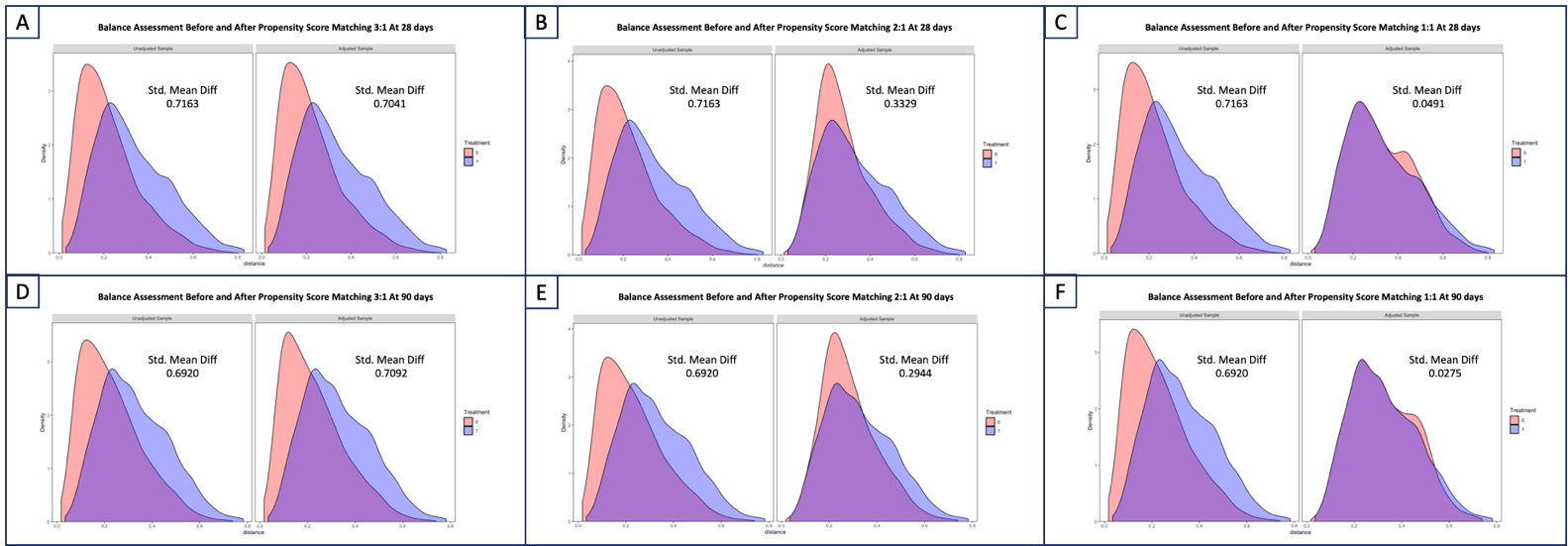


**Supplementary Table 1.**

| Supplementary Table 1. ICD-9 Code with description | |
| --- | --- |
| ICD9 CODE | **Description** |
| 48249 | Other Staphylococcus pneumonia |
| 48281 | Pneumonia due to anaerobes |
| 48282 | Pneumonia due to Escherichia coli [E. coli] |
| 48283 | Pneumonia due to other gram-negative bacteria |
| 4800 | Pneumonia due to adenovirus |
| 4801 | Pneumonia due to respiratory syncytial virus |
| 4802 | Pneumonia due to parainfluenza virus |
| 4803 | Pneumonia due to SARS-associated coronavirus |
| 4808 | Pneumonia due to other viruses not elsewhere classified |
| 4809 | Viral pneumonia, unspecified |
| 481 | Pneumococcal pneumonia [Streptococcus pneumoniae pneumonia] |
| 4820 | Pneumonia due to Klebsiella pneumoniae |
| 4821 | Pneumonia due to Pseudomonas |
| 4822 | Pneumonia due to Hemophilus influenzae [H. influenzae] |
| 48230 | Pneumonia due to Streptococcus, unspecified |
| 48231 | Pneumonia due to Streptococcus, group A |
| 48232 | Pneumonia due to Streptococcus, group B |
| 48239 | Pneumonia due to other Streptococcus |
| 48240 | Pneumonia due to Staphylococcus, unspecified |
| 48241 | Methicillin is susceptible pneumonia due to Staphylococcus aureus |
| 48242 | Methicillin-resistant pneumonia due to Staphylococcus aureus |
| 48284 | Pneumonia due to Legionnaires' disease |
| 48289 | Pneumonia due to other specified bacteria |
| 4829 | Bacterial pneumonia, unspecified |
| 4830 | Pneumonia due to mycoplasma pneumoniae |
| 4831 | Pneumonia due to chlamydia |
| 4838 | Pneumonia due to other specified organism |
| 4841 | Pneumonia in cytomegalic inclusion disease |
| 4843 | Pneumonia in whooping cough |
| 4845 | Pneumonia in anthrax |
| 4846 | Pneumonia in aspergillosis |
| 4847 | Pneumonia in other systemic mycoses |
| 4848 | Pneumonia in other infectious diseases classified elsewhere |
| 485 | Bronchopneumonia, organism unspecified |
| 486 | Pneumonia, organism unspecified |
| 4870 | Influenza with pneumonia |
| 4871 | Influenza with other respiratory manifestations |
| 4878 | Influenza with other manifestations |
| 48801 | Influenza due to identified avian influenza virus with pneumonia |
| 48802 | Influenza due to identified avian influenza virus with other respiratory manifestations |
| 48809 | Influenza due to identified avian influenza virus with other manifestations |
| 48811 | Influenza due to identified 2009 H1N1 influenza virus with pneumonia |
| 48812 | Influenza due to identified 2009 H1N1 influenza virus with other respiratory manifestations |
| 48819 | Influenza due to identified 2009 H1N1 influenza virus with other manifestations |
| 48881 | Influenza due to identified novel influenza A virus with pneumonia |
| 48882 | Influenza due to identified novel influenza A virus with other respiratory manifestations |
| 48889 | Influenza due to identified novel influenza A virus with other manifestations |

ICD-9: International Classification of Diseases 9^th^ Edition

**Supplementary Table 2.** Age-Stratified Sensitivity Analysis of Statin Use and Its Association with Mortality

| **Age group: 18-55 years old (n=1011), mean in treated vs. untreated** | **Unmatched cohort** | | | | **Matched cohort** | | | |  |
| --- | --- | --- | --- | --- | --- | --- | --- | --- | --- |
|  | **No statin, (n=891)** | **Statin,**  **(n= 120)** | **Standardised differences** | **p-value** | **No statin, (n=891)** | **Statin, (n=120)** | **Standardised differences** | **p-value** |  |
|  |  |  |  |  |  |  |  |  |  |
| Age | 43.5 | 47.8 | 0.502 | <0.001 | 46.6 | 47.7 | 12.8 | 0.282 |  |
| Charlson Comorbidity Index | 3.16 | 4.31 | 0.433 | <0.001 | 4.98 | 4.25 | -27.4 | 0.051 |  |
| Generalised Malignancy | 0.13 | 0.08 | -0.155 | 0.137 | 0.111 | 0.085 | -8.3 | 0.512 |  |
| Immunosuppression | 0.12 | 0.13 | -0.054 | 0.585 | 0.170 | 0.119 | -15.4 | 0.268 |  |
| Acute Kidney Disease | 0.24 | 0.35 | 0.245 | 0.009 | 0.341 | 0.341 | 0.0 | 1.00 |  |
| Chronic Kidney Disease | 0.07 | 0.18 | 0.334 | <0.001 | 0.213 | 0.171 | -12.9 | 0.409 |  |
| Transplanted | 0.03 | 0.03 | -0.032 | 0.748 | 0.017 | 0.025 | 5.2 | 0.653 |  |
| Autoimmune Disease | 0.03 | 0.04 | 0.054 | 0.554 | 0.042 | 0.042 | 0.0 | 1.00 |  |
| SAPS II | 30.37 | 30.69 | 0.024 | 0.802 | 31.61 | 30.79 | -6.2 | 0.627 |  |
| SOFA | 4.84 | 4.95 | 0.031 | 0.753 | 4.74 | 4.97 | 6.6 | 0.590 |  |
| Creatinine at day 1 | 1.43 | 1.99 | 0.272 | 0.003 | 1.73 | 1.91 | 8.3 | 0.508 |  |
| GCS on day 1 | 13.78 | 13.90 | 0.045 | 0.655 | 13.88 | 13.94 | -2.0 | 0.869 |  |
| Temperature | 37.07 | 37.00 | -0.113 | 0.256 | 37.003 | 37 | -0.7 | 0.960 |  |
| Viral Pneumonia | 0.05 | 0.07 | 0.121 | 0.170 | 0.042 | 0.059 | 7.2 | 0.555 |  |
| ARDS | 0.47 | 0.57 | 0.201 | 0.039 | 0.564 | 0.572 | 1.7 | 0.896 |  |
| **Age group: 56-75 years old (n=1959), mean in treated vs. untreated** | **No statin, (n=1347)** | **Statin,**  **(n= 612)** | **Standardised differences** | **p-value** | **No statin, (n=1347)** | **Statin, (n=612)** | **Standardised differences** | **p-value** |  |
| Age | 65.35 | 66.45 | 0.194 | <0.001 | 66.35 | 66.42 | 1.1 | 0.850 |  |
| Charlson Comorbidity Index | 5.64 | 6.38 | 0.284 | <0.001 | 6.33 | 6.36 | 1.1 | 0.851 |  |
| Generalised Malignancy | 0.24 | 0.15 | -0.231 | <0.001 | 0.144 | 0.150 | 1.7 | 0.747 |  |
| Immunosuppression | 0.09 | 0.06 | -0.100 | 0.047 | 0.055 | 0.062 | 2.5 | 0.627 |  |
| Acute Kidney Disease | 0.33 | 0.39 | 0.111 | 0.022 | 0.386 | 0.386 | 0.0 | 1.00 |  |
| Chronic Kidney Disease | 0.16 | 0.25 | 0.208 | <0.001 | 0.252 | 0.245 | -1.6 | 0.791 |  |
| Transplanted | 0.02 | 0.02 | <0.001 | 0.999 | 0.009 | 0.016 | 6.2 | 0.315 |  |
| Autoimmune Disease | 0.04 | 0.03 | -0.043 | 0.388 | 0.021 | 0.029 | 4.6 | 0.363 |  |
| SAPS II | 39.19 | 39.43 | 0.019 | 0.701 | 39.85 | 39.42 | -3.3 | 0.555 |  |
| SOFA | 5.11 | 5.27 | 0.047 | 0.335 | 5.40 | 5.26 | -4.2 | 0.450 |  |
| Creatinine at day 1 | 1.46 | 1.86 | 0.246 | <0.001 | 1.92 | 1.84 | -4.4 | 0.513 |  |
| GCS on day 1 | 13.81 | 13.80 | -0.005 | 0.909 | 13.49 | 13.79 | 11.6 | 0.055 |  |
| Temperature | 36.90 | 36.92 | 0.034 | 0.483 | 36.91 | 36.91 | 1.0 | 0.853 |  |
| Viral Pneumonia | 0.04 | 0.04 | 0.019 | 0.696 | 0.044 | 0.039 | -2.6 | 0.668 |  |
| ARDS | 0.50 | 0.53 | 0.062 | 0.202 | 0.495 | 0.526 | 6.2 | 0.277 |  |
| **Age group: >75 years old (n=1483), mean in treated vs. untreated** | **No statin, (n=1009)** | **Statin,**  **(n=474)** | **Standardised differences** | **p-value** | **No statin, (n=1009)** | **Statin, (n=474)** | **Standardised differences** | **p-value** |  |
| Age | 83.49 | 83.88 | -0.130 | 0.020 | 82.6 | 82.8 | 6.3 | 0.313 |  |
| Charlson Comorbidity Index | 6.91 | 7.80 | 0.382 | <0.001 | 7.8 | 7.7 | -4.2 | 0.540 |  |
| Generalised Malignancy | 0.14 | 0.13 | -0.047 | 0.403 | 0.13 | 0.12 | -1.2 | 0.847 |  |
| Immunosuppression | 0.03 | 0.02 | -0.058 | 0.361 | 0.02 | 0.02 | 1.3 | 0.826 |  |
| Acute Kidney Disease | 0.43 | 0.42 | -0.017 | 0.755 | 0.44 | 0.42 | -3.9 | 0.555 |  |
| Chronic Kidney Disease | 0.26 | 0.40 | 0.295 | <0.001 | 0.40 | 0.39 | -1.8 | 0.791 |  |
| Autoimmune Disease | 0.01 | 0.01 | -0.019 | 0.736 | 0.010 | 0.012 | 1.8 | 0.762 |  |
| SAPS II | 45.35 | 44.04 | -0.106 | 0.060 | 45.26 | 44.07 | -9.5 | 0.139 |  |
| SOFA | 5.33 | 4.94 | -0.126 | 0.028 | 5.38 | 4.94 | -14.2 | 0.022 |  |
| Creatinine at day 1 | 1.51 | 1.64 | 0.106 | 0.058 | 1.59 | 1.62 | 2.3 | 0.694 |  |
| GCS on day 1 | 13.12 | 13.56 | 0.158 | 0.005 | 13.41 | 13.56 | 5.4 | 0.379 |  |
| Temperature | 36.77 | 36.78 | 0.011 | 0.844 | 36.75 | 36.77 | 4.8 | 0.437 |  |
| Viral Pneumonia | 0.03 | 0.04 | 0.045 | 0.412 | 0.029 | 0.040 | 5.7 | 0.376 |  |
| ARDS | 0.49 | 0.46 | -0.055 | 0.311 | 0.449 | 0.459 | 2.1 | 0.744 |  |
| **Abbreviations:** Simplified Acute Physiology Score II (SAPS II), Sequential Organ Failure Assessment score (SOFA), Glasgow Coma Scale (GCS), Acute Respiratory Distress Syndrome (ARDS). | | | | | | | | |  |

**Supplementary Table 3.** Sensitivity Analysis: Estimates of Treatment Effects on Mortality

| **Method** | **ATE** | **Standard Error** | **z Value** | **p Value** | **95% CI** |
| --- | --- | --- | --- | --- | --- |
| **Age group: 18-55 years old (n=1011)** | | | | | |
| Propensity Score Matching | -0.008 | 0.054 | -0.16 | 0.871 | [-0.116, 0.098] |
| Inverse-Probability Weighting | 0.023 | 0.049 | 0.47 | 0.638 | [-0.073, 0.119] |
| **Age group: 56-75 years old (n=1959)** | | | | | |
| Propensity Score Matching | -0.102 | 0.028 | -3.53 | <0.001 | [-0.158, -0.045] |
| Inverse-Probability Weighting | -0.093 | 0.023 | -4.00 | <0.001 | [-0.139, -0.047] |
| **Age group: >75 years old (n=)** | | | | | |
| Propensity Score Matching | -0.119 | 0.031 | -3.81 | <0.001 | [-0.180, -0.057] |
| Inverse-Probability Weighting | -0.107 | 0.027 | -3.92 | <0.001 | [-0.161, -0.053] |
| **Abbreviations:** Average Treatment Effect (ATE), Confidence Intervale (CI). | | | | | |

**Supplementary Table 4.** Sensitivity Analysis of Statin and Macrolide Use and Its Association with Mortality

| **Macrolide antibiotics = 1 (n=1458), mean in treated vs. untreated** | **Unmatched cohort** | | | | **Matched cohort** | | | |  |
| --- | --- | --- | --- | --- | --- | --- | --- | --- | --- |
|  | **No statin, (n=430)** | **Statin,**  **(n= 1028)** | **Standardised differences** | **p-value** | **No statin, (n=430)** | **Statin, (n=1028)** | **Standardised differences** | **p-value** |  |
|  |  |  |  |  |  |  |  |  |  |
| Age | 63.5 | 71.1 | 0.491 | <0.001 | 71 | 71.07 | 0.5 | 0.943 |  |
| Charlson Comorbidity Index | 5.10 | 6.80 | 0.605 | <0.001 | 6.67 | 6.76 | 3.5 | 0.595 |  |
| Generalised Malignancy | 0.13 | 0.11 | -0.059 | 0.314 | 0.114 | 0.086 | 8.5 | 0.173 |  |
| Immunosuppression | 0.11 | 0.06 | -0.054 | 0.003 | 0.067 | 0.056 | -4.3 | 0.479 |  |
| Acute Kidney Disease | 0.35 | 0.45 | 0.213 | <0.001 | 0.478 | 0.450 | -5.8 | 0.411 |  |
| Chronic Kidney Disease | 0.16 | 0.33 | 0.396 | <0.001 | 0.334 | 0.331 | -0.6 | 0.942 |  |
| Transplanted | 0.01 | 0.02 | 0.048 | 0.384 | 0.025 | 0.018 | -5.6 | 0.487 |  |
| Autoimmune Disease | 0.03 | 0.01 | -0.094 | 0.126 | 0.014 | 0.014 | 0.0 | 1.00 |  |
| SAPS II | 36.31 | 36.76 | 0.199 | 0.001 | 39.13 | 39.30 | 1.3 | 0.830 |  |
| SOFA | 4.82 | 4.92 | 0.033 | 0.567 | 4.92 | 4.92 | -0.1 | 0.990 |  |
| Creatinine at day 1 | 1.39 | 1.84 | 0.285 | <0.001 | 1.85 | 1.82 | -1.5 | 0.851 |  |
| GCS on day 1 | 13.86 | 13.95 | 0.038 | 0.525 | 13.63 | 13.95 | 14.2 | 0.036 |  |
| Temperature | 36.93 | 36.88 | -0.092 | 0.120 | 36.90 | 36.88 | -3.2 | 0.615 |  |
| Viral Pneumonia | 0.06 | 0.07 | 0.060 | 0.288 | 0.074 | 0.072 | -0.9 | 0.896 |  |
| ARDS | 0.43 | 0.52 | -0.020 | 0.728 | 0.432 | 0.516 | 16.8 | 0.014 |  |
| **Macrolide antibiotics = 0 (n=3074), mean in treated vs. untreated** | **No statin, (n=2298)** | **Statin,**  **(n=776)** | **Standardised differences** | **p-value** | **No statin, (n=2298)** | **Statin, (n=776)** | **Standardised differences** | **p-value** |  |
| Age | 64.91 | 71.00 | 0.422 | <0.001 | 71.47 | 70.97 | -3.5 | 0.454 |  |
| Charlson Comorbidity Index | 5.39 | 6.69 | 0.460 | <0.001 | 6.79 | 6.67 | -4.3 | 0.389 |  |
| Generalised Malignancy | 0.20 | 0.15 | -0.142 | 0.001 | 0.179 | 0.147 | -8.5 | 0.086 |  |
| Immunosuppression | 0.07 | 0.05 | -0.100 | 0.021 | 0.032 | 0.050 | 7.5 | 0.074 |  |
| Acute Kidney Disease | 0.33 | 0.37 | 0.076 | 0.066 | 0.344 | 0.368 | 4.9 | 0.340 |  |
| Chronic Kidney Disease | 0.17 | 0.28 | 0.278 | <0.001 | 0.253 | 0.279 | 6.3 | 0.250 |  |
| Transplanted | 0.02 | 0.01 | -0.092 | 0.045 | 0.003 | 0.006 | 2.4 | 0.479 |  |
| Autoimmune Disease | 0.03 | 0.03 | 0.008 | 0.845 | 0.018 | 0.029 | 6.9 | 0.134 |  |
| SAPS II | 39.30 | 40.96 | 0.118 | 0.005 | 40.69 | 40.96 | 1.9 | 0.701 |  |
| SOFA | 5.24 | 5.21 | -0.007 | 0.874 | 5.18 | 5.21 | 1.1 | 0.828 |  |
| Creatinine at day 1 | 1.49 | 1.75 | 0.165 | <0.001 | 1.59 | 1.75 | 10.4 | 0.033 |  |
| GCS on day 1 | 13.46 | 13.59 | 0.043 | 0.298 | 13.52 | 13.58 | 2.0 | 0.686 |  |
| Temperature | 36.89 | 36.86 | -0.048 | 0.253 | 36.85 | 36.86 | 0.4 | 0.931 |  |
| Viral Pneumonia | 0.03 | 0.03 | 0.001 | 0.990 | 0.034 | 0.027 | -4.8 | 0.379 |  |
| ARDS | 0.47 | 0.50 | 0.063 | 0.131 | 0.501 | 0.498 | -0.5 | 0.919 |  |
| **Abbreviations:** Simplified Acute Physiology Score II (SAPS II), Sequential Organ Failure Assessment score (SOFA), Glasgow Coma Scale (GCS), Acute Respiratory Distress Syndrome (ARDS). | | | | | | | | |  |

**Supplementary Table 5.** Sensitivity Analysis: Estimates of Treatment Effects on Mortality according to Macrolide use

| **Method** | **ATE** | **Standard Error** | **z Value** | **p Value** | **95% CI** |
| --- | --- | --- | --- | --- | --- |
| **Macrolide antibiotics = 1 (n=1458)** | | | | | |
| Propensity Score Matching | -0.015 | 0.034 | -0.44 | 0.659 | [-0.082, 0.051] |
| Inverse-Probability Weighting | -0.055 | 0.028 | -1.97 | 0.049 | [-0.111, 0.440] |
| **Macrolide antibiotics = 0 (n=3074)** | | | | | |
| Propensity Score Matching | -0.094 | 0.025 | -3.65 | <0.001 | [-0.144, -0.043] |
| Inverse-Probability Weighting | -0.090 | 0.020 | -4.40 | <0.001 | [-0.130, -0.050] |
| **Abbreviations:** Average Treatment Effect (ATE), Confidence Intervale (CI). | | | | | |
